# Connectomic analysis of Alzheimer’s disease using percolation theory

**DOI:** 10.1162/netn_a_00221

**Published:** 2022-02-01

**Authors:** Parker Kotlarz, Juan C. Nino, Marcelo Febo

**Affiliations:** Department of Materials Science and Engineering, University of Florida, Gainesville, FL, USA; Department of Psychiatry, University of Florida, Gainesville, FL, USA

**Keywords:** Alzheimer’s disease, Connectomics, Percolation theory, Biomarker, fMRI, Graph theory

## Abstract

Alzheimer’s disease (AD) is a severe neurodegenerative disorder that affects a growing worldwide elderly population. Identification of brain functional biomarkers is expected to help determine preclinical stages for targeted mechanistic studies and development of therapeutic interventions to deter disease progression. Connectomic analysis, a graph theory–based methodology used in the analysis of brain-derived connectivity matrices was used in conjunction with percolation theory targeted attack model to investigate the network effects of AD-related amyloid deposition. We used matrices derived from resting-state functional magnetic resonance imaging collected on mice with extracellular amyloidosis (TgCRND8 mice, *n* = 17) and control littermates (*n* = 17). Global, nodal, spatial, and percolation-based analysis was performed comparing AD and control mice. These data indicate a short-term compensatory response to neurodegeneration in the AD brain via a strongly connected core network with highly vulnerable or disconnected hubs. Targeted attacks demonstrated a greater vulnerability of AD brains to all types of attacks and identified progression models to mimic AD brain functional connectivity through betweenness centrality and collective influence metrics. Furthermore, both spatial analysis and percolation theory identified a key disconnect between the anterior brain of the AD mice to the rest of the brain network.

## Author Summary

Accurate biomarkers of Alzheimer’s disease (AD) are needed for early diagnosis and treatments. Connectomic analysis, a graph theory approach, coupled with percolation theory, a network attack approach, were applied here to analyze neuroimaging through a quantitative lens. We report a marker of AD vulnerability, which highlighted a core network disconnected from key hubs, notably within the anterior portion of the brain disconnected. Additionally, preliminary models using targeted attacks provide potential pathways of neurodegeneration from the control state to the diseased state. These findings show key differences in brain connectivity due to AD and provide a potential methodology for identifying biomarkers.

## INTRODUCTION

Alzheimer’s disease (AD) is a neurodegenerative disorder that accounts for 60%–80% of cases of dementia with typical symptoms involving memory loss, confusion, changes in personality, social withdrawal, and language difficulties (2020 Alzheimer’s Disease Facts and Figures, 2020). Sporadic nonfamilial forms of AD affect 5.8 million people in the United States aged 65 years or older, and this population is estimated to rise to about 13.8 million by 2050 (Hebert et al., 2013). Major risk factors for familial AD include genetics (Bertram et al., 2010; Farrer et al., 1997) and previous family history (Mayeux et al., 1991; Yi et al., 2018), while age and apolipoprotein-E status are risk factors for sporadic late-onset AD that accounts for over 95% of all cases (Hebert et al., 2013; Koffie et al., 2012; Launer et al., 1999). Due to AD’s progressive nature, different stages of AD show variations in the severity of symptoms. For instance, preclinical AD entails measurable brain functional changes with few or no symptoms, mild cognitive impairment (MCI) with more pronounced brain alterations and mild symptoms, and AD with dementia with significant symptomology and brain structural changes (Sperling et al., 2011). Through this progression model, identification of features that distinguish preclinical AD from other stages is important for timely therapeutic intervention and the targeting of stage-specific biological factors that may delay the progression of AD and its symptoms (Bakker et al., 2015; Crous-Bou et al., 2017). Identifying biomarkers in patients who are likely to develop AD through cerebrospinal fluid (CSF) tests and radioligand-based neuroimaging techniques are serving as a major clinical resource to understand and treat AD before it progresses significantly (Frisoni et al., 2017). However, CSF tests are invasive for patients, have the possibility of introducing infection into the central nervous system, and cannot always be performed. In addition, positron emission tomography neuroimaging techniques can serve as a more clinically applicable approach for identifying biomarkers, but these are limited by the amount of exposure to radiolabeled compounds. Conversely, magnetic resonance (MRI)-based biomarkers, particularly using approaches that interrogate the brain’s white matter and functional connectomic patterns, are highly valuable due to their safe, noninvasive nature, and because there are several national and international data repositories that can be used to investigate lead biomarkers that have clinical importance.

One encouraging approach at identifying potential biomarkers in AD is through the field of functional connectomics. Major advancements in brain mapping techniques, such as functional MRI (fMRI), allow for comprehensive and quantitative assessments of functional connectivity in the human brain (Milano et al., 2019). Functional connectivity measures the co-activation between the activity of different brain regions (Friston, 1994). Functional connectomics uses data derived from imaging modalities to create adjacency matrices (networks of nodes and edges), or connectomes, that are then analyzed with broadly applicable mathematical principles of graph theory (Rubinov & Sporns, 2010). Through network analysis, a growing range of quantifiers can be explored to understand local and global brain connectivity patterns. Connectomic analysis can also be applied to compare differences between brains through statistical (Shehzad et al., 2014), quantifier-based (Pompilus et al., 2020), and machine learning techniques (Sarwar et al., 2021). Previous functional connectomic analysis in rodent models have found anatomical motifs (Díaz-Parra et al., 2017), rich-club organization (Liang et al., 2018), and contextual changes in network topology (Pompilus et al., 2020). Additionally, connectomics has yielded promising results in understanding and identifying biomarkers in AD with an increasing focus on utilizing functional connectivity (Alderson et al., 2018; Brier et al., 2012; Damoiseaux et al., 2012; Filippi et al., 2020; Khazaee et al., 2015; Ren et al., 2020; Ye et al., 2019).

**Figure 1.**
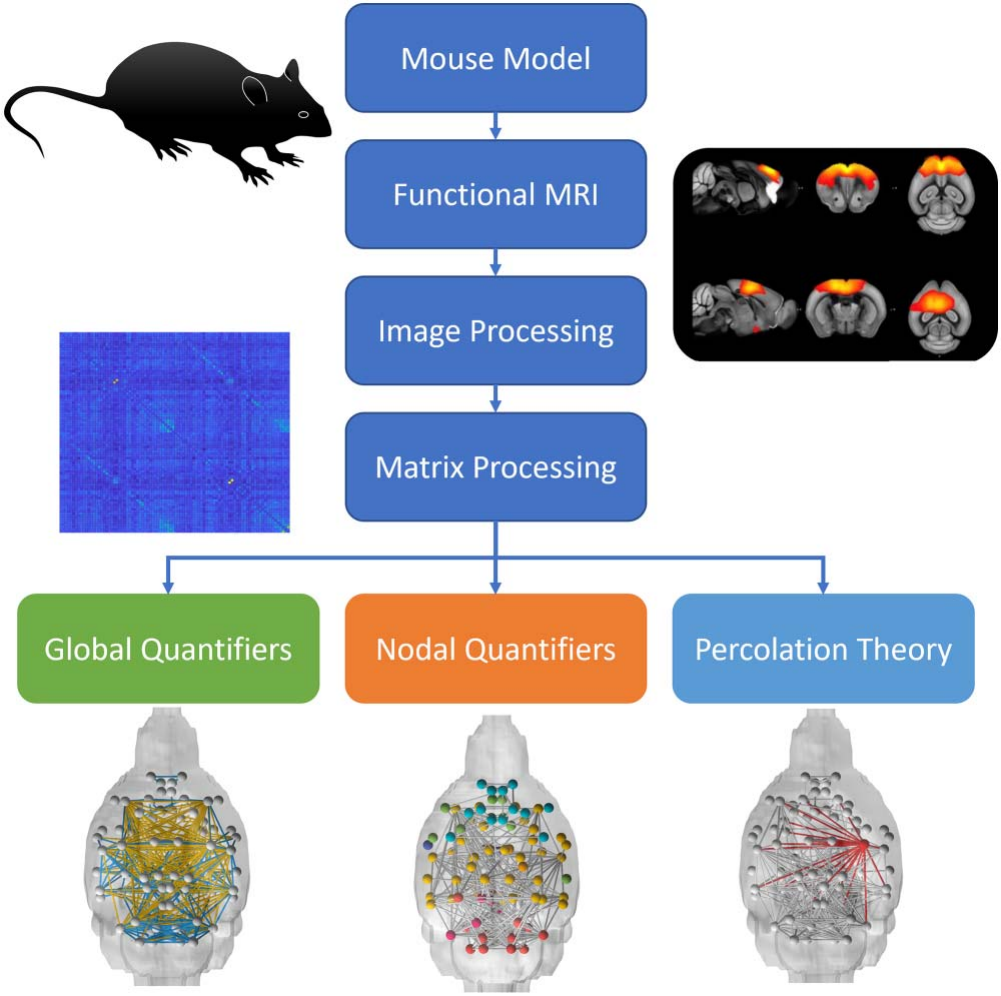
Overview of connectomic analysis.

In the present study we used a network analysis approach based on percolation theory, a subbranch of graph theory that involves removing (or adding) nodes or edges to assess their importance or influence on overall network integrity (Albert & Barabási, 2002). Percolation theory has been proposed as a model to determine network resilience and possibly inform on the stages of neurodegenerative progression of diseases such as AD (Fornito et al., 2015; Lo et al., 2015). Percolations model either random network failure via indiscriminate deletions of individual nodes or edges, or it can use a planned or targeted attack strategy in which node removal is guided by quantifiers that rank node prominence within the network. Through these random and targeted attacks, the resilience of the network can be measured through quantifiers reflecting network integrity (largest cluster size) (Albert et al., 2000) and communication efficiency (path length) (Achard et al., 2006; Kaiser et al., 2007). Given evidence of disrupted synaptic communication in amyloid mouse models and functional connectivity in the presence of high amyloid load in AD (Myers et al., 2014), information on network resilience through the use of percolation theory may provide a robust biomarker for early detection and progression in AD. Such models of network resilience are currently underdeveloped in AD (Mrdjen et al., 2019), but their optimization and testing may lead to the discovery of key network epicenters as identified in AD propagation studies (Zhou et al., 2012). In the present proof-of-concept study, we applied graph theory to analyze functional connectivity networks derived from the TgCRND8 mouse model of amyloidosis and control mice of the same background strain (Colon-Perez et al., 2019) (Figure 1). In addition to providing a spatial neuroanatomical analysis of the distribution of network quantifiers, we used percolation theory to investigate network resilience as a function of amyloid status.

## METHODS

### Description of Node-Based Functional Connectivity Data

The functional connectivity adjacency matrices analyzed in the present work were part of a larger study originally published by Colon-Perez et al. (2019). Also, the methods used for image acquisition and image processing, the brain regions included in the present analysis, and the generation of the adjacency matrices were published in the original study (Colon-Perez et al., 2019). Therefore, no new data were collected for the present study, which focuses on novel analyses of resting-state fMRI data collected previously. Briefly, in the prior original study, TgCRND8 mice were bred via transgenic (Tg) males (carrying amyloid precursor protein) with C57B6/C3H F1 females (Envigo) (Janus et al., 2000). Additionally, mice were subject to intracerebroventricular adeno-associated viral vector interleukin-6 (IL6) injections at P0, which led to brain expression of murine IL6 or enhanced green fluorescent protein as described in Chakrabarty et al. (2010). In this original study by Colon-Perez et al. (2019), fMRI images were collected through a single-shot spin-echo echo planar imaging sequence (TE = 15 ms, TR = 2 s, 180 repetitions, 15 × 11 mm in plane, 14 slices with 0.9-mm thickness per slice, data matrix = 64 × 48) using a 11.1 T MRI scanner. fMRI image processing included (1) removal of time series spikes, (2) slice timing correction, (3) motion and linear drift correction, (4) subject-to-atlas registration, (5) regression of white matter and CSF (ventricle) signals, (6) bandpass filtering (0.01–0.1 Hz), and (7) spatial blurring (0.3 mm FWHM). Finally, average BOLD signals were extracted based on their atlas location, creating 4,005 pairwise Pearson correlation coefficients.

We used a subset of data collected on mice with extracellular amyloidosis (TgCRND8 mice, *n* = 17, male = 6, female = 11) and control littermates (*n* = 17, male = 9, female = 8). For clarity of data presentation, we refer to the TgCRND8 mouse as ‘AD mice’ and nontransgenic mice as ‘control mice’ throughout the manuscript. As described in the original study, TgCRND8 mice have early-onset expression of human mutant APP (Swedish APP KM670/671NL and Indiana APP V717F), which increases human APP five times above endogenous murine APP. These mice have cognitive impairment, Aβ plaque deposits, and increased inflammation at 3–4 months of age, synaptic deficits, and some synaptic and neuronal loss in the hippocampus by 6 months. (Chishti et al., 2001). The mice were 8 months old at the time of imaging. Subsets of mice had additional experimental manipulations such as viral vector administration into the brain at postnatal day 0 and increased brain expression of the cytokine, IL6. However, we focus on the distinction between the two main groups to investigate the ability of network quantifiers to discern differences as a function of amyloid status.

### Matrix Manipulation and Processing

To address subject-specific differences in the location of nodes with strong and weak connections across matrices, which can impact results even after controlling for graph density, we first assessed general characteristics of the targeted attack approach in group-averaged matrices and then conducted separate statistical comparisons on quantifiers drawn from individually prethresholded matrices. For group-averaged comparisons, weighted matrices were first averaged within each group and then normalized and thresholded at 10% graph density. This threshold removed weak and negative edges and kept small-world characteristics during group comparisons. A 10% graph density was chosen, as thresholds below 10% produced networks that were too fragmented to differentiate and upper graph densities (>10%) showed similar trends as 10% but with differences less apparent. This group-averaged approach highlighted stable and strong connections that were generally consistent with connections in individual-level analyses and thus served as a representative model for each group. While group-averaged comparisons have been utilized in previous studies, (Brown et al., 2012; Hagmann et al., 2008; Perry et al., 2015; Smith et al., 2013), it is important to note that individual-specific weak connections will unavoidably be omitted from analyses (Amico & Goñi, 2018; Gordon et al., 2017; Roberts et al., 2017).

### Network Quantifier and Statistical Analysis

Weighted global and nodal quantifiers were applied to both the individual and group-averaged matrices. Global quantifiers examine aspects of the network as a whole. Largest cluster size and graph density were calculated to understand network composition. Measures of integration that analyze how network information flows efficiently include characteristic path length (CPL) (Watts & Strogatz, 1998), network radius, network diameter, and global efficiency (Latora & Marchiori, 2001). CPL is characterized by the average shortest path, or the number of edges, between all the nodes in the network (Watts & Strogatz, 1998). This quantifier details possible routes of transmission of information of data in the network by measuring functional integration of the network, with smaller CPLs indicating a more integrated network. Global efficiency (Latora & Marchiori, 2001), the inverse of CPL, also measures integration and may be more valuable in disconnected networks, like those observed in the present study, due to the ability of this quantifier to account for disconnected nodes (Rubinov & Sporns, 2010). For CPL, global efficiency, network radius, and network diameter, diagonal and infinite distances were omitted to prevent infinite values since thresholding and attacking the networks create network fragments. Measures of segregation that examine how the network is separated into different functional modules or groups include maximized modularity (Newman, 2006) and transitivity (Newman, 2003). Assortativity (Leung & Chau, 2007; Newman, 2002), a measure of resilience, was also calculated. Nodal quantifiers examine individual nodes and their role in the network. Quantifiers that measure nodal characteristics include degree centrality (DC) measuring the number of connections and strength (ST) which measures the total sum of edge weights connected to a node. Nodal measures of segregation examine how individual nodes function within groups include clustering coefficient (CC) (Onnela et al., 2005; Watts & Strogatz, 1998) and local efficiency (LE) (Latora & Marchiori, 2001). Measures of centrality which explore how nodes interact with the rest of the brain include eigenvector centrality (EC) (Newman, 2016), participation coefficient (PC) (Guimerà & Amaral, 2005), and betweenness centrality (BC) (Freeman, 1978). The quantifiers above were calculated using MATLAB’s Brain Connectivity Toolbox (MATLAB. Version 9.7.0.1319299, R2019b; Rubinov & Sporns, 2010), and a detailed list of equations and derived information from the associated measures above can be found extensively detailed from Rubinov and Sporns (2010). In addition to the previously stated quantifiers, collective influence and small worldness were also calculated. Collective influence (CI), a novel quantifier that ranks nodes by using an optimized percolation method designed to break up major network components (Morone et al., 2016; Morone & Makse, 2015), was calculated using ComplexCi in C++ (F. Zhu, 2018) and transferred to MATLAB for further analysis. Additionally, small worldness (Humphries & Gurney, 2008), a global measure that identifies high clustering with a short CPL compared to a random network, was also calculated using MATLAB.

Statistical analysis was conducted to compare control and AD mouse networks. Global and nodal quantifiers were examined across groups. Using JMP (JMP, Version Macintosh; SAS Institute Inc., Cary, NC, 1989–2019), the normality of the data was examined. Since different quantifiers displayed different degrees of normality, especially in the nodal group quantifiers, the Wilcoxon rank-sum test was used to test for statistical significance since it is a more conservative approach that does not require the assumption of normality. Furthermore, using nonparametric tests such as the Wilcoxon rank-sum test is shown to be more appropriate for smaller studies (Fagerland, 2012). This test does not require normality and is also resistant to outliers. Effect sizes (r), calculated by dividing the test statistic by the square root of the number of samples, are also reported for significant values.

### Spatial Analysis Using Brain Net Viewer

Nodal quantifiers were compared regionally using nodes generated using a mouse brain parcellation and template (Moore et al., 2016). Spatial analysis through BrainNet Viewer (Xia et al., 2013), a graphical tool that overlays network nodes and connections on 3D brain models, was used to visualize the group-average control and AD mouse brains. To analyze shared connections, the weighted averaged matrices were binarized and then subtracted to create a matrix to input into BrainNet Viewer. Through BrainNet Viewer, between-region and within-region brain connectivity were analyzed and specific modules, nodes, and connections were highlighted.

### Network Targeted Attacks and Progression Analysis

The control and AD mouse networks were subsequently analyzed using targeted node removal involving selectively removing nodes from the connectivity matrix by changing all connections for that node to zero based on a specific nodal quantifier until the network was completely degraded. After each attack, global and nodal network quantifiers were calculated for the new network to analyze for progressive change. There were two categories of attack methods: basis and iterative. Basis attacks used the initially calculated quantifier of the unaltered matrix to remove nodes. Iterative attacks continually recalculated the quantifier on the new network after each node removal. Strength, degree centrality, betweenness centrality, eigenvector centrality, clustering coefficient, local efficiency, and participation coefficient were included using both basis and iterative attack schemes. Collective influence was utilized only as a basis attack scheme due to its high computational cost. Additionally, a random attack scheme was also conducted that removed nodes at random. A limitation to both the random and basis attack schemes involves removing nodes that already are disconnected. Since these attack schemes do not continually update like the iterative attack schemes, they have the possibility of removing a node that has already previously been disconnected. This limitation did not appear to have a significant effect on our results. The response to different attack schemes was compared between control and AD mice. Furthermore, attack schemes in healthy mice were compared to initial quantifiers in AD mice to examine if any attack schemes model disease development.

## RESULTS

### Global Network Characteristics

Global network measures were determined for both individual and group-averaged matrices. Table 1 summarizes the global network quantifiers for control and AD mice. No differences between these groups were observed in either individual or group-averaged network quantifiers (i.e., quantifiers from group-averaged matrices were within bounds of quantifiers analyzed across individual subjects; see Table 1). This result provided evidence of consistency between individual and group-wise analyses and suggested that the latter accurately represents network properties. A difference in betweenness centrality was observed between the networks of individual AD mice (113.72) and the group-average brain of the same group (44.18). This difference most likely stems from an emphasis on stronger and consistent stable connections across subjects (a ‘core’ network) by preaveraging individual matrices prior to calculating network quantifiers. Within groups, there were also no significant differences between male and female functional networks.

**Table 1.** Global network quantifiers across groups

Quantifier	Control (Ind.)	Control (Avg.)	Alzheimer (Ind.)	Alzheimer (Avg.)	*P* value
Small worldness	1.81 ± 0.0844 (0.348)	1.43	1.76 ± 0.111 (0.457)	2.07	0.877
Modularity	0.374 ± 0.0208 (0.0856)	0.381	0.356 ± 0.0255 (0.105)	0.361	0.642
Transitivity	0.113 ± 0.0159 (0.0654)	0.0684	0.163 ± 0.0275 (0.113)	0.151	0.185
Largest cluster size	83.8 ± 2.916 (12.0)	81.0	80.8 ± 3.29 (13.6)	62.0	0.514
CPL	11.7 ± 0.889 (3.66)	20.4	8.40 ± 0.753 (3.12)	7.19	0.0138*
Global efficiency	0.117 ± 0.0123 (0.0509)	0.0667	0.167 ± 0.0197 (0.0811)	0.164	0.0138*
Radius	13.1 ± 2.03 (8.35)	2.89	9.22 ± 1.68 (6.92)	1.07	0.0943
Diameter	26.4 ± 1.71 (7.06)	64.8	19.3 ± 1.72 (7.08)	16.5	0.0045**
Assortativity	0.221 ± 0.0334 (0.138)	0.246	0.195 ± 0.0367 (0.151)	−0.0146	0.438
Avg. CC	0.0875 ± 0.0107 (0.0441)	0.0560	0.116 ± 0.0154 (0.0635)	0.120	0.134
Avg. ST	2.23 ± 0.179 (0.737)	1.42	3.05 ± 0.266 (1.10)	2.62	0.0167*
Avg. BC	127 ± 9.55 (39.4)	151	113 ± 9.97 (41.1)	44.2	0.301
Avg. EC	0.0683 ± 0.00232 (0.00958)	0.0669	0.0728 ± 0.00329 (0.0136)	0.0718	0.459
Avg. LE	0.117 ± 0.0124 (0.0509)	0.0753	0.152 ± 0.0161 (0.0663)	0.150	0.0820
Avg. PC	0.357 ± 0.0270 (0.111)	0.306	0.362 ± 0.0334 (0.138)	0.128	0.931

*Note*. Data are presented as mean ± standard error. Standard deviation values are shown in parentheses. Wilcoxon’s rank-sum test used to compare control vs. AD mice. CPL = characteristic path length; CC = clustering coefficient; ST = strength; BC = betweenness centrality; EC = eigenvector centrality; LE = local efficiency; PC = participation coefficient. Ind, individual analysis = indicative of quantifiers calculated on individual subject matrices. Avg, group-average analysis = indicative of quantifiers calculated after subject matrices were first averaged.

* *p* < 0.05.

** *p* < 0.01.

There were significant differences in CPL (*p* = 0.0138, *r* = 0.419), efficiency (*p* = 0.0138, *r* = 0.419), diameter (*p* = 0.0045, *r* = 0.484), and average strength (*p* = 0.0167, *r* = 0.408) between control and AD mice. In the group-average groups, there were notable differences in the same quantifiers and also in largest cluster size, assortativity, and average betweenness centrality. For all groups, the average degree was 8.911 with a graph density of 0.1001 as a result of the 10% graph density thresholding method. Both group average and individuals as a group of both control and AD brain networks had a heavy-tailed degree distribution with the group-averaged diseased brain containing a significantly longer tail.

### Brain Connectivity Characteristics

#### Nodal network characteristics.

**Figure 2.**
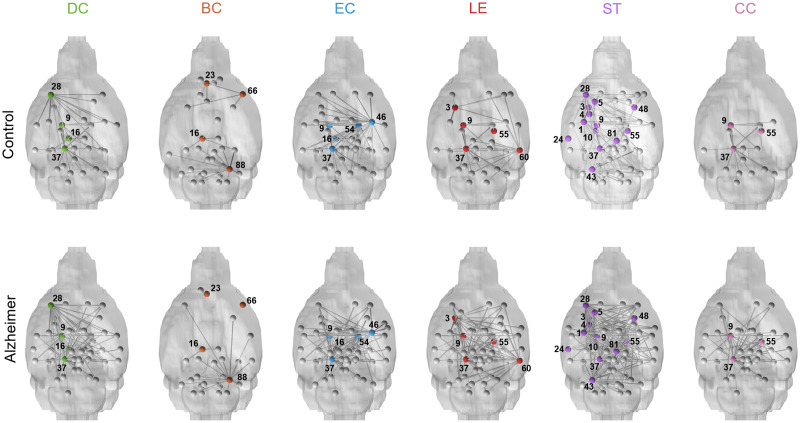
Nodes with significant differences in nodal quantifiers between control and AD mice. Nodes are highlighted in color on representative 3D mouse brains, and their existing connections to other nodes are shown (in gray). DC = degree centrality; BC = betweenness centrality; EC = eigenvector centrality; LE = local efficiency; ST = strength; CC = clustering coefficient; 1 = left amygdala; 3 = left dorsal striatum; 4 = left globus pallidus; 5 = left ventral pallidus; 9 = left ventral thalamic tier; 10 = left lateral tier of the thalamus; 16 = left retrosplenium; 23 = left prelimbic cortex; 24 = left auditory cortex; 28 = left rostral piriform cortex; 37 = left mesencephalic reticular formation; 43 = left cerebellar peduncle; 46 = right amygdala; 48 = right dorsal striatum; 54 = right ventral thalamic tier; 55 = right lateral tier of the thalamus; 60 = right entorhinal; 66 = right rostral insular cortex; 81 = right ventral tegmental area; 88 = right cerebellar peduncle.

Table 2 summarizes the anatomical locations of nodes with significant differences in nodal quantifiers between control and AD mice with nodes visualized in Figure 2. There were no significant differences between nodal quantifiers for individualized and group-averaged brains for each respective group. No nodes had significant differences in participation coefficients. Notably, both the left ventral thalamic tier and mesencephalic reticular formation nodes had significant differences in all nodal quantifiers except for betweenness centrality. Other nodes that were significantly different in multiple categories included the left retrosplenium, left rostral piriform cortex, left dorsal striatum, and right lateral tier of the thalamus. In their respective groups, the left and right sides of the brains had very few significant differences: betweenness centrality in AD cerebellar peduncle (*p* = 0.0371, *r* = 0.354), betweenness centrality in control mouse amygdala (*p* = 0.0403, *r* = 0.349), clustering coefficient in AD entorhinal (*p* = 0.494, *r* = 0.334), and degree centrality in AD mouse substantia nigra (*p* = 0.0412, *r* = 0.347).

**Table 2.** Node locations with significant differences in nodal quantifiers

Nodal location	Control (Ind.)	Control (Avg.)	Alzheimer (Ind.)	Alzheimer (Avg.)	*P* value (Ind.)	Effect size
Degree centrality						
Ventral thalamic tier L.	6.94 ± 1.12 (4.60)	4	13.1 ± 1.79 (7.37)	18	0.0093**	0.443
Retrosplenium L.	10.4 ± 1.19 (4.91)	4	6.41 ±1.33 (5.47)	2	0.0171*	0.406
Piriform rostral L.	9.29 ± 2.12 (8.73)	12	13.0 ± 2.28 (9.38)	20	0.0431*	0.344
Mesencephalic Reticular formation L.	10.4 ± 1.85 (7.61)	13	18.2 ± 2.27 (9.38)	37	0.0107*	0.435
Betweenness centrality						
Retrosplenium L.	171.65 ± 35.14 (144.88)	6	77.178 ± 21.017 (86.89)	0	0.0375*	0.354
Prelimbic L.	125.76 ± 24.00 (98.91)	576	56.24 ± 17.67 (72.84)	0	0.0164*	0.408
Insular rostral R.	160.59 ± 38.48 (158.65)	530	60.82 ± 15.50 (63.91)	0	0.0305*	0.368
Cerebellar peduncle R.	286.24 ± 36.23 (149.39)	152	172.82 ± 35.50 (146.38)	192	0.0287*	0.372
Eigenvector centrality						
Ventral thalamic tier L.	0.0421 ± 0.0122 (0.0505)	0.0213	0.126 ± 0.0190 (0.0748)	0.159	0.0036**	0.496
Retrosplenium L.	0.0770 ± 0.0196 (0.0810)	0.0236	0.0430 ± 0.0164 (0.0676)	1.36E-29	0.0494*	0.334
Mesencephalic reticular formation L.	0.0751 ± 0.0214 (0.0882)	0.0974	0.152 ± 0.0241 (0.0992)	0.252	0.0138*	0.419
Amygdala R.	0.0721 ± 0.0193 (0.0794)	0.0931	0.124 ± 0.0198 (0.0814)	0.196	0.0476*	0.337
Ventral thalamic tier R.	0.0561 ± 0.0152 (0.0628)	0.0588	0.124 ± 0.0231 (0.0953)	0.183	0.0439*	0.343
Local efficiency						
Dorsal striatum L.	0.0944 ± 0.0254 (0.105)	0.0805	0.187 ± 0.0375 (0.155)	0.178	0.0456*	0.340
Ventral thalamic tier L.	0.0818 ± 0.0212 (0.0874)	0.123	0.218 ± 0.0369 (0.152)	0.234	0.0083**	0.450
Mesencephalic reticular formation L.	0.116 ± 0.0275 (0.114)	0.0834	0.223 ± 0.0362 (0.149)	0.177	0.0125*	0.425
Lateral tier thalamus R.	0.0694 ± 0.0179 (0.0737)	0	0.180 ± 0.0406 (0.167)	0.240	0.0114*	0.431
Entorhinal R.	0.162 ± 0.0234 (0.0966)	0.0982	0.243 ± 0.0308 (0.127)	0.202	0.496*	0.334
Strength						
Amygdala L.	2.47 ± 0.563 (2.32)	1.37	5.68 ± 1.32 (5.46)	7.02	0.0476*	0.337
Dorsal striatum L.	1.66 ± 0.448 (1.85)	0.708	4.18 ± 1.09 (4.51)	5.07	0.0093**	0.443
Globus pallidus L.	0.968 ± 0.266 (1.10)	0.114	2.64 ± 0.735 (3.03)	1.78	0.0300*	0.369
Ventral pallidus L.	0.953 ± 0.182 (0.752)	0.131	1.69 ± 0.258 (1.06)	0.526	0.0341*	0.360
Ventral thalamic tier L.	1.66 ± 0.389 (1.60)	0.574	5.02 ± 1.16 (4.78)	5.24	0.0029**	0.508
Lateral tier thalamus L.	1.19 ± 0.228 (0.941)	0.487	2.55 ± 0.487 (2.01)	1.18	0.0201*	0.396
Auditory L.	1.24 ± 0.255 (1.05)	0	2.77 ± 0.823 (3.39)	1.73	0.0476*	0.337
Piriform rostral L.	2.48 ± 0.754 (3.11)	1.78	5.07 ± 1.44 (5.93)	5.44	0.0287*	0.372
Mesencephalic reticular formation L.	2.78 ± 0.699 (2.88)	1.74	6.93 ± 1.38 (5.70)	10.7	0.0062**	0.467
Cerebellar peduncle L.	3.62 ± 0.426 (1.76)	3.63	6.11 ± 0.836 (3.45)	8.53	0.0241*	0.384
Dorsal striatum R.	1.78 ± 0.426 (1.76)	0.63	3.72 ± 0.942 (3.88)	3.67	0.0167*	0.408
Lateral tier thalamus R.	1.22 ± 0.207 (0.855)	0.114	2.49 ± 0.509 (2.10)	1.44	0.0210*	0.393
Ventral tegmental area R.	1.40 ± 0.211 (0.869)	0.795	2.75 ± 0.508 (2.09)	1.52	0.0371*	0.355
Clustering coefficient						
Ventral thalamic tier L.	0.0575 ± 0.0149 (0.0615)	0.0999	0.174 ± 0.0324 (0.134)	0.178	0.0055**	0.473
Mesencephalic reticular formation L.	0.0835 ± 0.0226 (0.0933)	0.0548	0.154 ± 0.0879 (0.129)	0.0850	0.0341*	0.360
Lateral tier thalamus R.	0.0504 ± 0.0133 (0.0549)	0	0.144 ± 0.0380 (0.157)	0.223	0.0242*	0.383

*Note*. Data are shown as mean ± standard error (standard deviation). Wilcoxon rank-sum test was used to compare control and AD mice. L = left; R = right. Ind., individual analysis = indicative of quantifiers calculated on individual subject matrices. Avg., group-average analysis = indicative of quantifiers calculated after subject matrices were first averaged.

* *p* < 0.05.

** *p* < 0.01.

#### Connectivity hubs and patterns.

**Figure 3.**
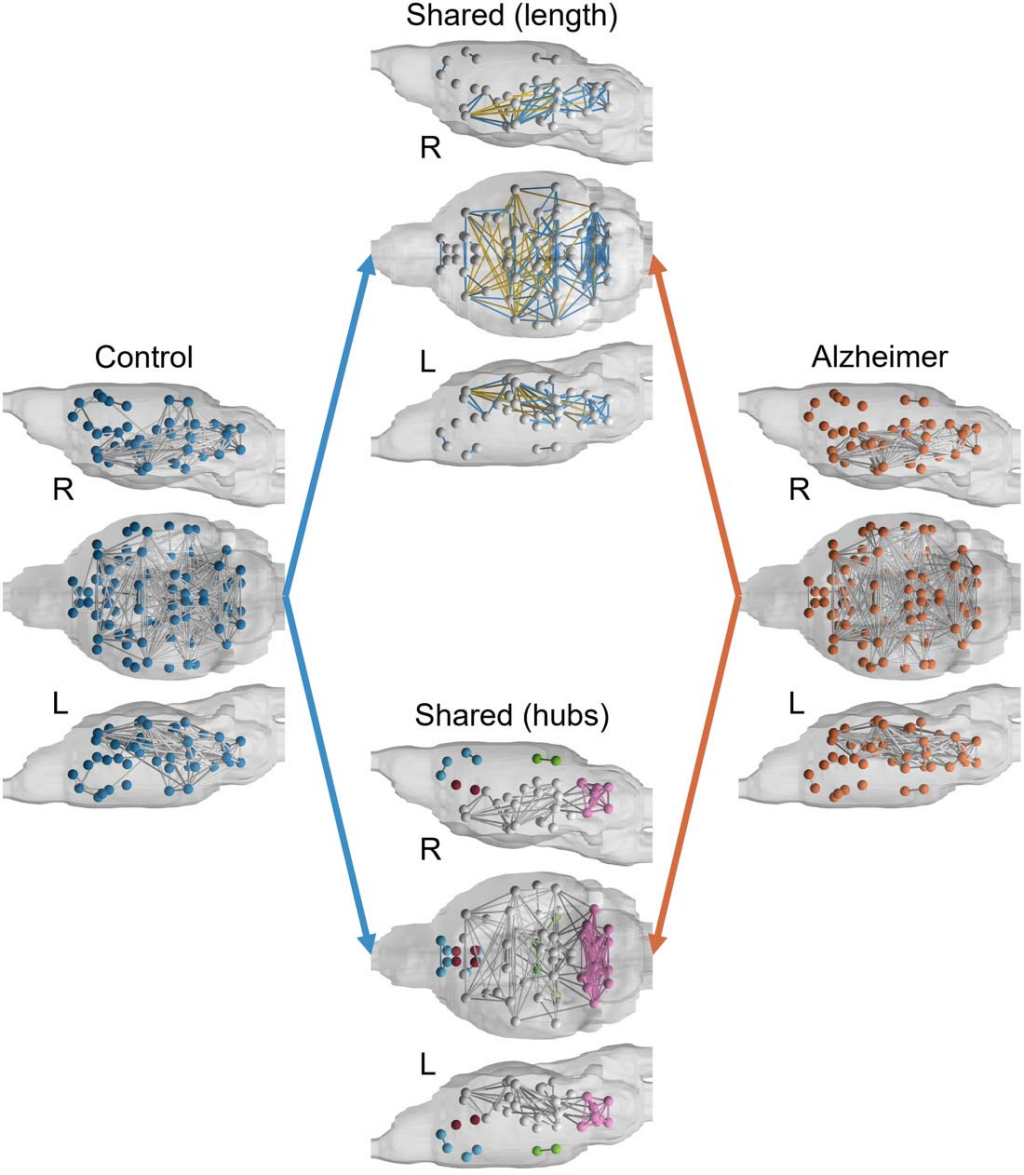
Binarized connectome maps of control and AD mice. Shown in the middle panel (top) are short- and long-range connections common to both groups (nodes with no connections are not shown). Also shown in the middle panel (bottom) are nodes representing hubs that are common between the two groups. In the shared-length brain, yellow connections represent long-range connections while blue connections indicate close-range connections. L = left; R = right.

Binarized group-averaged brain networks were mapped onto mouse brains in Figure 3 along with shared connections. Shared connections demonstrate a core network that is present in both brains. A notable difference between brains is the lack of connections between the prefrontal cortex and the rest of the AD mouse brain that are present in the controls. This is further demonstrated in the shared-hub brain model, which shows three sets of disconnected modules that are only connected in the control brain model: left and right retrosplenium, left and right visual cortex (in green); left and right septum, left and right infralimbic area (in red); left and right anterior cingulate, left and right orbital cortex, left and right prelimbic area, left secondary motor cortex (M2) since right M2 is completely disconnected (in blue). In the shared network, both control and AD brains show a highly connected module in the hindbrain: left and right inferior colliculus, left and right second cerebellar lobule, left and right cerebellar simple lobule, left and right third cerebellar lobule, left and right cerebellar peduncle, left and right fifth cerebellar lobule, and left and right areas that include deep cerebellar nuclei (cerebellar nuclear area; in pink). Furthermore, the shared connections in the brain, excluding the highly connected posterior module, are long-range transhemispheric connections (in yellow).

In terms of connectivity strength, both groups showed very strong intrahemispheric connections between anterior nodes, including right prelimbic and right orbital nodes (both groups had a connection strength of 1 indicating the strongest connection) and left prelimbic and left orbital nodes (control: 0.7263; AD: 0.6748). Both groups also had left and right midline posterior thalamic tier connected to each other but separated from the rest of the brain, with a notably stronger self-connection in AD mice than in controls (control: 0.3462; AD: 0.9326). Similarly, both groups showed strong connections between the cerebellar peduncle and cerebellar nuclear area in their respective hemisphere with minimal difference between controls and AD networks (left control: 0.4512; left AD: 0.7695; right control: 0.6427; right AD: 0.612).

### Targeted Attack of Brain Networks

#### Brain resilience against attack schemes.

**Figure 4.**
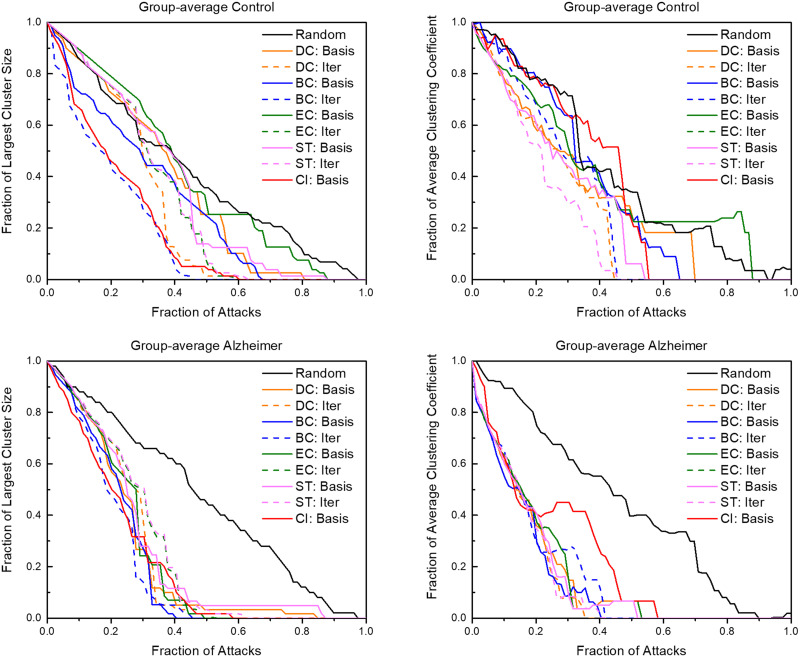
The graphs above show the reactions of control and AD brain networks in response to targeted attacks. The axes are normalized for comparison. “Basis” indicates attack schemes that use quantifiers calculated with the unaltered network while “Iter” indicates attack schemes that iteratively calculate the attack quantifier after each attack. DC = degree centrality; BC = betweenness centrality; EC = eigenvector centrality; ST = strength; CI = collective influence.

The group-averaged brain networks of control and AD mice responded different to the various targeted attack schemes (Figure 4). On average, iterative attacks degraded both brain networks at a faster rate than basis attacks. Iterative betweenness centrality and collective influence attacks degraded the network at the quickest rate in both groups. Within the first 20% of nodes removed, both brains were reduced to only 40% of the original cluster size. When examining the largest cluster size of a network, the control group had a wider variety of degradation as opposed to the AD group in which all attacks degraded the network at a significantly faster rate than random attacks. This variety of degradation was also seen in examining attacks compared to the average clustering coefficient (data not shown). Similarly, this pattern of greater resilience to degradation was also recognizable in examining attacks compared to betweenness centrality, average degree, number of edges remaining, average local efficiency, and average strength to a lesser degree. Notably, the clustering coefficient was affected at a rate comparable to random in iterative betweenness centrality and collective influence, even at the quick degradation rate in the largest cluster size of the control group. Iterative and basis attacks based on clustering coefficient, local efficiency, and participation coefficient were also explored but showed results consistent with a reduced efficiency at degrading the network compared to the shown attack schemes.

#### Control brain networks compared to AD baseline.

**Figure 5.**
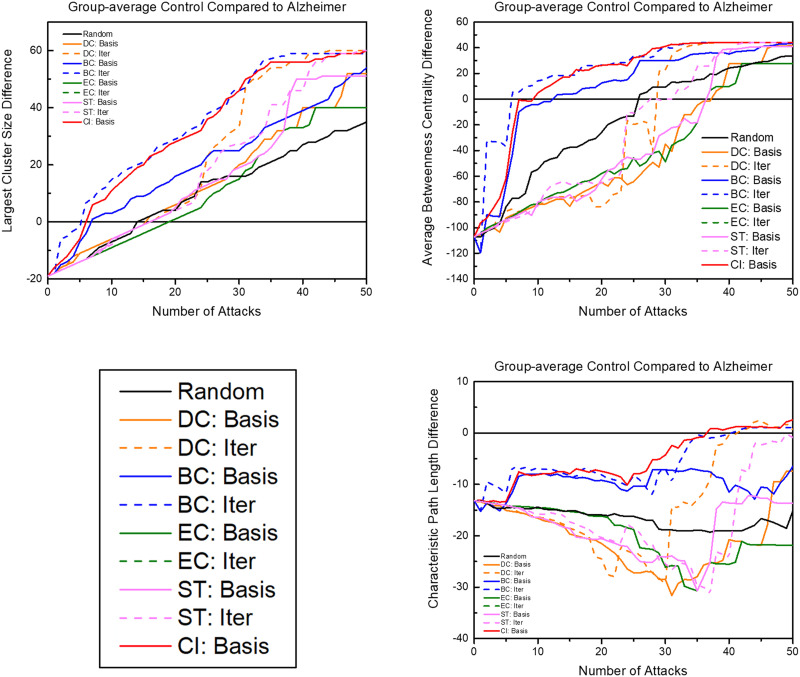
The graphs above show the progression of the control network compared to the AD baseline (diseased quantifier at baseline – healthy quantifier after each attack). The number of attacks axis shortened to 50 rather than extended to removing the full 90 nodes since, after a certain number of attacks, quantifiers become unstable due to the small and disconnected set of networks in the brains. “Basis” indicates attack schemes that use quantifiers calculated with the unaltered network, while “Iter” indicates attack schemes that iteratively calculate the attack quantifier after each attack. DC = degree centrality; BC = betweenness centrality; EC = eigenvector centrality; ST = strength; CI = collective influence.

To examine the progression of the degradation effects of node attacks on AD functional networks, quantifiers examined during attacks to the group-averaged control brains were compared to quantifiers of the unaltered group-averaged AD brain (Figure 5). Iterative betweenness centrality and collective influence attacks reached the largest cluster size baseline in between attacks five and six. When examining these attack schemes compared to the average betweenness centrality, which had a large difference in global measures (Table 1), the control brain reached the level of betweenness centrality of the diseased brain in only six attacks for iterative betweenness centrality and seven attacks for collective influence. In contrast, it takes other attack schemes 16 to 38 attacks to reach the level of betweenness centralized of the diseased baseline. Since the goal is to identify biomarkers, early network degradation is prioritized. Regarding characteristic path length, both betweenness centrality attack schemes and collective influence are the only attack schemes to progressively move closer to the AD baseline without regressing further away. For both transitivity and network diameter, collective influence was the only measure that progressively moved close to matching the diseased brain baseline, while iterative betweenness centrality stays at the healthy baseline and then quickly reached the diseased baseline.

**Figure 6.**
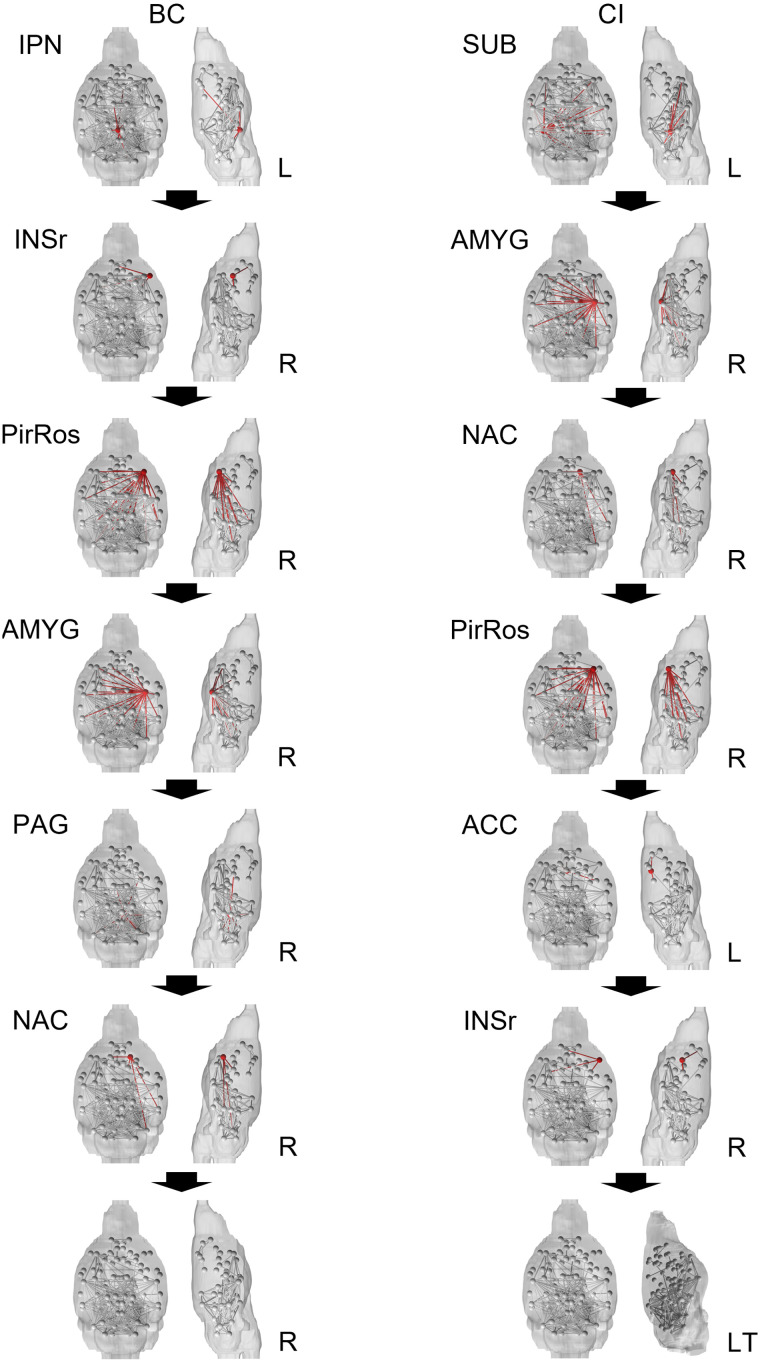
The brains above show the healthy group-averaged brain progression through the iterative betweenness centrality and collective influence attack schemes. The red node and accompanying connections highlighted is the node that will be removed next. After two attacks in the BC scheme, the anterior portion of the control brain networks was disconnected. After six attacks in the CI scheme, the anterior portion of the control brain was disconnected; the side view of the brain was tilted to better illustrate this. BC = betweenness centrality, CI = collective influence; IPN = interpeduncular nucleus; INSr = rostral insular cortex; PirRos = rostral piriform cortex; AMYG = amygdala; PAG = periaqueductal gray; NAC = accumbens; SUB = subiculum; ACC = anterior cingulate cortex; L = left; LT = left tilt; R = right.

Similarities were found when exploring the iterative betweenness centrality and collective influence attack schemes more closely. Since both attacks reached the largest cluster size in between five and six attacks, the first six attacks were mapped onto mice brains in Figure 6. The context of the attacks reaching the AD baseline of betweenness centrality diseased baseline in very few attacks (six for iterative betweenness centrality and seven for collective influence) is also important. In the first six attacks, iterative betweenness centrality and collective influence shared four nodes: right rostral piriform cortex, right amygdala, right rostral insular cortex, and right accumbens. In collective influence’s seventh attack, the attack at which it reached diseased baseline betweenness centrality, the right periaqueductal gray node is shared with iterative betweenness centrality’s fifth attack.

Both attack schemes also fractured the network significantly, separating key nodes from the main network. After only two iterative betweenness centrality attacks or six collective influence attacks, key anterior nodes were completely disconnected from the rest of the brain: left and right orbital cortex, left and right prelimbic area, left and right primary somatosensory cortex, and left secondary motor region. For iterative betweenness centrality after two attacks, other key anterior nodes were disconnected: right primary motor cortex, right secondary motor cortex, and left and right anterior cingulate cortex. These nodes, excluding the left anterior cingulate cortex, which was removed in the fifth collective influence attack, are still connected in the collective influence scheme by a single connection of the right anterior cingulate cortex to the left interpeduncular nucleus. After six iterative betweenness centrality attacks, the left and right infralimbic, left and right septum, and right dorsal striatum were fully disconnected. These nodes remained connected in the collective influence attack scheme after six attacks but were subsequently disconnected following the seventh attack through the removal of the right periaqueductal gray node.

## DISCUSSION

The observed global network differences between control and amyloid mice are possibly due to Aβ’s pathological effect on synaptic transmission, which in turn may impact brain-wide functional connectivity. Both individual and group-averaged networks of control mice had significantly higher characteristic path length and lower global efficiency than AD mice (Table 1). While some functional connectome studies have reported opposite findings (Stam et al., 2007; J. Wang et al., 2013; R. Wang et al., 2014) or no significant differences, such as a similar mouse model study (Brier et al., 2014; Kesler et al., 2018; Seo et al., 2013; Supekar et al., 2008), other studies have found similar results (Palesi et al., 2016; Sanz-Arigita et al., 2010). This variation in results most likely stems from a difference in MR image acquisition and signal processing approaches, which can impact network topology and graph sparsity. Relatedly, compared to AD mice, both individual and group-averaged controls had a larger network diameter, which measures the longest possible path between nodes in the network. These differences in CPL, global efficiency, and diameter may be associated with the observed reductions in functional connectivity of rostral regions of the brain to caudal areas in AD mice. While shown in the group-averaged control mice having a higher largest cluster size and as shown through the more connected hubs in Figure 3, these findings also indicate a similar pattern of a core network with many disconnected nodes found in individual control and AD brain networks. For instance, in human AD, differences in connectivity between anterior portions or posteromedial regions of the default mode network have been associated with mood disorders such as depression, amyloid deposition, and cognitive impairment (Andrews-Hanna et al., 2010; Coutinho et al., 2016; Mormino et al., 2011; X. Zhu et al., 2012). This rostral-caudal imbalance in connectivity patterns might be an important marker of an underlying AD pathology. Even with comparable largest cluster sizes when looking at individual subjects, the differences in these quantifiers point toward a more centralized connectivity scheme in AD mice as opposed to the more distant connectivity pattern formed as a result of stronger connections to distant hubs in control mice. This is further supported by the lower average strength in both control mice and higher BC in the group-averaged AD mice, indicating overall weaker connections between distant hubs of a core network. Additional quantifier analysis of a compensatory response, neuroanatomical localization of functional changes, and diaschisis can be found in the Supporting Information.

Examining similarities and differences in brain resilience in response to targeted attacks can further elucidate critical changes in the AD brain. Targeted attacks degrading both brain networks at a faster rate than random is consistent with findings due to the brains having heavy-tailed distributions (Albert et al., 2000) (Figure 4). Since the AD brain heavy-tailed distribution was extended, this may explain the higher susceptibility of the diseased brain to all attacks demonstrated by all attacks performing significantly better than random attacks. The extended heavy-tailed distribution may be a result of the brain compensating to AD by relying more heavily on high-strength nodes to minimize wiring cost to hubs (Crossley et al., 2014). Similarly, the negative assortativity (Table 1) of the group-averaged AD brain also indicates a low-resilience network with vulnerable high-degree hubs (Newman, 2002). The effectiveness of the collective influence and iterative BC further support the degradation of high-strength connecting hub nodes since these measures specifically examine hub-to-hub connectivity. AD brains having lower resilience to targeted attacks is supported by earlier studies showing AD-affected areas are more vulnerable (Klupp et al., 2014; Myers et al., 2014). This increased vulnerability demonstrates the possibility of the compensatory response only effectively delaying cognitive function degradation in the short-term and may be a factor in the transition from MCI to AD.

Part of the effectiveness of a biomarker may be determined by how early it can detect pathological signs before severe disease progression, exploring the earliest stages of degradation in unaffected brains compared to AD brains has a high potential to be identifiable before the onset of AD. Collective influence and iterative BC attacks were found to be the most likely AD progression model by comparing these attacks to an AD baseline (Figure 5). As demonstrated by quantifier, spatial, and targeted attack analyses, the AD brains showed a markedly different hub connectivity pattern in their greater reliance on high-strength connecting hub nodes, long-range connectivity patterns, and increased connectivity in the core network. This hub-pattern weakness in AD brains is also shown by the decreased BC in the group-averaged AD brain (Table 1). Other studies have also found a significant decrease in BC in AD brains (Cai et al., 2019; D. Wang et al., 2020) and thereby provide a potential explanation for the feeder-hub connectivity differences. This relationship is also supported by classification schemes identifying BC as an important difference between AD and healthy brains (Jalili, 2017; Sheng et al., 2019). Since collective influence is optimized to separate hubs, it is also similarly related to BC and the hub-connectivity pattern shown in AD.

Through these quantifiers, two progression models were mapped onto mouse brains (Figure 6). Eight nodes that were removed were identified using these progression models that were present early in network degradation: left interpeduncular nucleus, right insular cortex, right piriform cortex, right amygdala, right periqueductal gray, right accumbens, left subiculum, and left anterior cingulate cortex. The right amygdala and right rostral insula were also identified when analyzing differences between individual brains through BC and eigenvector centrality, respectively (Figure 2). While the removal of these nodes may model disease progression, it is equally important to look at the nodes disconnected from the brain through these targeted attacks. Both attack schemes (six BC attacks, seven CI attacks) disconnected the following nodes from the core network: left and right orbital, left and right prelimbic, left and right primary somatosensory cortex, left secondary motor region, the left and right infralimbic cortex, left and right septal nucleus, and right dorsal striatum. After two BC attacks the right primary and secondary motor regions and the anterior cingulate were also disconnected from the main network. These nodes are still connected in the CI attack scheme through a single connection of the right anterior cingulate with the left interpeduncular nucleus. The left prelimbic and right dorsal striatum were also identified when analyzing differences between individual brains through BC and node strength, respectively (Figure 2). Most notably, many of these nodes were found in disconnected hubs in Figure 3. These include the left and right septum, left and right infralimbic cortex, left and right anterior cingulate cortex (BC only), left and right orbital, left and right prelimbic, left m2, and right m2 (BC only). Both the BC and CI attack schemes quickly disconnect the anterior portion of the brain with the rest of the brain while maintaining within-region connectivity. Previous evidence of anterior-to-posterior disconnections in AD has been found in both graph theory and biological studies (Dauwan et al., 2016; Delbeuck et al., 2003; Kocagoncu et al., 2020; Liu et al., 2014). Since connections between anterior nodes were some of the strongest in the network, the anterior-to-posterior disconnect could explain cognitive deficits in AD patients rather than degradation in the anterior brain. Namely, communication between anterior regions playing important roles in active behavioral outputs and processes such as decision-making and cognitive flexibility and posterior regions involved in long-term memory retrieval may be adversely affected in AD, according to our present results. Furthermore, these regions identified both by spatial analysis and percolation theory could act as potential biomarker regions to track early onset of AD pathway degradation. Additionally, the importance of the two related measures of betweenness centrality and collective influence underscore the effect AD has on disconnecting key hubs from the core network.

This study does have some limitations. Mice were scanned while under isoflurane anesthesia and results may need comparisons with other sedatives, such as dexmedetomidine, in order to determine generalizability of the present results. The mouse model is highly specific for a set of mutations involved in dominant inherited AD, but the majority of cases (>95%) are spontaneous late-onset AD, with no clear mechanism in sight, yet. ApoE alleles have been found to be risk factor, but the others are age and comorbidity with cardiovascular conditions. Thresholding at 10% graph density may also play a significant role in how the brain networks are constructed (Roberts et al., 2017) and the effect of thresholding should be further examined. A clear limitation is also the use of a threshold based on fMRI connectivity but not based on true anatomical connectivity. This is a limitation and a reason why in other cases labs use multiple thresholds to quantify the stability/consistency of the connectomic measures across thresholds. Additionally, as mentioned in the Methods, the group-averaging method to find group-averaged healthy and diseased brains may ignore subtle individual-level connections that are important (Amico & Goñi, 2018; Gordon et al., 2017; Roberts et al., 2017). Individual-level connections should also be explored. Finally, the connectomic approach in general must be scrutinized. While quantifiers may characterize global estimates of connectivity and topology, the underlying neurobiological drivers are not fully explained by this approach.

This study utilizes connectomics to analyze differences in control and AD mouse brains. Through quantifier, spatial, and targeted attack analyses, a short-term compensatory response was found in the core of the AD brain through a vulnerable high-strength hub connector reliant network. Furthermore, a significant disconnect between hubs and the core network was present in the AD brain. This was notably reinforced using the collective influence and betweenness centrality progression models showing an anterior-to-posterior disconnect that may explain cognitive deficits through a disconnection syndrome lens. These preliminary progression models also identified key nodes that can potentially serve as biomarkers to identify early connectivity changes in patients with a risk of developing AD. Together, these results point toward a progression model identifying key brain regions that serve as anterior-to-posterior connecting nodes that may explain AD symptomology and act as a biomarker for early intervention.

## SUPPORTING INFORMATION

Supporting information for this article is available at https://www.doi.org/10.1162/netn_a_00221.

## AUTHOR CONTRIBUTIONS

Parker Kotlarz: Conceptualization; Data curation; Formal analysis; Investigation; Methodology; Software; Validation; Visualization; Writing – original draft; Writing – review & editing. Juan C. Nino: Conceptualization; Funding acquisition; Project administration; Resources; Supervision; Writing – review & editing. Marcelo Febo: Conceptualization; Data curation; Funding acquisition; Project administration; Resources; Supervision; Writing – original draft; Writing – review & editing.

## FUNDING INFORMATION

Parker Kotlarz, University of Florida AI2020 Catalyst Grant, Award ID: AWD09459. Marcelo Febo, Foundation for the National Institutes of Health (https://dx.doi.org/10.13039/100000009), Award ID: AG065819. Marcelo Febo, National Science Foundation (https://dx.doi.org/10.13039/501100008982), Award ID: DMR-1644779.

## TECHNICAL TERMS

Connectomics: Graph theory–based methodology that involves deconstructing the brain into mathematical matrices.
Functional connectivity: Measure of the coactivation of different brain regions.
Graph theory: Mathematical approach to studying relationships between structures (nodes) through their connections (edges).
Quantifier: Mathematical descriptor based on a relationship in the network.
Percolation theory: Subbranch of graph theory that analyzes the removal of nodes/edges of a network.
Graph density: Measure of how many overall connections are present out of possible connections in a network.
Group-averaged: Method of averaging matrices together to eliminate weak edges while keeping core network characteristics.
Small-world: Organized network whereby any node can reach another node in a short sequence of edges.
Betweenness centrality: Quantifies how often a node lies within a shortest path of the network.
Collective influence: Optimal percolation method through targeting region connecting nodes.
