# Supplementary material for: Connectomic analysis of Alzheimer’s disease using percolation theory
Source: Netw Neurosci. 2022 Feb 1;6(1):213–33. doi: 10.1162/netn_a_00221 (PMC9810282; doi:10.1162/netn_a_00221)
Supplement: Supplementary file 1 [file netn-06-213-s001.pdf]

Kotlarz, P., Nino, J. C. & Febo, M. (2022). Supporting information for "Connectomic Analysis of Alzheimer's Disease using Percolation Theory." *Network Neuroscience*, *6*(1), 213–233. https://doi.org/10.1162/netn_a_00221

Connectomic Analysis of Alzheimer's Disease using Percolation Theory

Parker Kotlarz[a], *Juan C. Nino[a], and *Marcelo Febo[b]

[a] Department of Materials Science and Engineering, University of Florida, Gainesville, Florida 32611

[b] Department of Psychiatry, University of Florida, Gainesville, Florida 32611

*J. Nino and M. Febo are co-senior authors

5. ADDITIONAL DISCUSSIONS

5.1. Compensatory Response

While the previous explanation stems from analyzing the destructive aspect of neuropathological changes in AD through the disruption of connections to distant modules in AD mice, another equally valid explanation is through compensation in response to neurodegeneration. Compensation of functional connectivity and underlying neuronal activity is suggested in previous research (Fornito et al., 2015). One study found an increased recruitment of existing 'neural' resources despite high amyloid-beta plaque burden, which helped deter cognitive decline (Elman et al., 2014). Other studies have also found evidence of hyperactivity at the onset of AD (Busche & Konnerth, 2015), with one *in vivo* mouse model study classifying 21% of neurons near amyloid plaques as hyperactive (Busche et al., 2008). This hyperactivity in the presence of beta-amyloid may also explain the lower CPL, higher global efficiency, and higher average strength observed in AD mice in the present study. One could posit that small core or localized 'pockets' of hyperactivity might produce high CPL values than control brains characterized by more distant connections. Furthermore, previous research indicates that the observed initial hyperactivity during cognitive tasks in early AD eventually progresses to reduced activity (perhaps connectivity) and declines in cognitive function (Grady et al., 2003; Sperling, 2007).

5.2. Neuroanatomical Localization of Functional Changes

Nodal differences between control and AD mice represent region-specific differences and can help pinpoint the neuroanatomical location of functional changes. As shown in **Error! Reference source not found.** and visualized in **Error! Reference source not found.**, the left ventral thalamic tier and mesencephalic reticular formation nodes had significant differences in degree centrality, eigenvector centrality, local efficiency, strength, and clustering coefficient. These regions are known to take part in processing and modulating sensory information coming from peripheral synaptic inputs. The reticular formation in particular has extensive ascending noradrenergic projections to the cortex with the potential capacity to coordinate activity across large scale cortical networks. Notably, these nodes do not have significant differences in BC. Since the AD nodes are significantly more connected than healthy brains, these nodes may be acting in a compensatory response due to degradation of other neural pathways. Other notable nodes with significant differences in multiple categories include the left retrosplenium, left piriform cortex, left dorsal striatum, and right lateral tier of the thalamus. Interestingly, the striatum receives widespread cortical afferents, as do lateral regions of the thalamus. The importance of these nodes could be related to compensatory functional gains in their role as cortical output regions in AD mice. Taken together with the above findings, it is possible that loss of 'filtering' functions in the ventral thalamic regions (e.g., altered activity of local interneurons) and an increased input activity to the cortex channels back larger amounts of output information through the lateral thalamic regions and the striatum. There is evidence for selective degeneration of inhibitory gamma amino butyric neurons in human AD (Kurucu et al., 2021), and these neurons also play important 'filtering' functions in thalamocortical networks. This could implicate the involvement of other connected areas, such as regions of cerebellar nuclei as well (as discussed below). The retrosplenial

cortex is known for its role in long-term memory and its connections to sensory regions of the cortex (Fournier et al., 2020). The piriform also plays a role in higher level stages of olfactory processing and olfactory-related memory functions (Bolding et al., 2020). It is unclear how amyloidosis might affect these regions in the AD mice. As noted previously, BC was higher in both individual matrices and group-averaged matrices of control mice than in the AD group. While examining nodal differences, BC is the only category in which controls consistently have a higher value than the AD group. This points toward the degradation of connecting nodes in AD mice since BC highlights nodes that connect different modules (Rubinov & Sporns, 2010). The nodes with this configuration included the left retrosplenium, left prelimbic, right insula, and right cerebellar peduncle. Three of these nodes (excluding the right cerebellar peduncle) are completely disconnected in AD brains. Due to their high BC in controls and absence in AD mice, these nodes may indicate specific regions that are detrimentally affected by amyloid burden or effects of this extracellular protein up or downstream from these regions.

5.3. Diaschisis

While differences between specific nodes may help to determine functional differences linked to regional amyloid plaque load, it is also important to consider diaschisis. This is defined as regions distant from the brain lesion that experience dysfunction (von Monakow, 1969). Diaschisis has been reported in functional connectivity networks of brain lesion models (Carrera & Tononi, 2014) and AD (Akiyama et al., 1989). This is further reinforced by observations of hypometabolism in regions with low amyloid-beta plaques (Klupp et al., 2014). The possibility of diaschisis playing a major role in AD dysfunction further emphasizes the importance of not only the specific nodal region but also the connectivity within and between regions. Three main

modules were observed to be connected to the rest of the brain in the group-average control group (**Error! Reference source not found.**). The first module includes the left and right retrosplenium and left and right primary visual cortex, suggesting a module dedicated to visual-spatial sensory processing. The second module included the left and right septum and left and right infralimbic cortex, areas involved in memory and emotion regulation. The third module includes the left and right anterior cingulate, left and right orbital and prelimbic cortices, and the secondary motor cortex, which collectively play roles in reward-seeking and general motivated behaviors as well as higher cognitive functions such as decision making. The second and third modules are located in the anterior portion of the brain. In both control and AD mice, the connection between the prelimbic and orbital nodes in their respective hemispheres was among the strongest connections in the brain. These strong connections suggest that while the anterior brain is strongly connected with itself in the AD brain, it is disconnected from other caudal brain areas. This further suggests an anterior-to-posterior disconnection model of AD rather than localized degradation of activity in the prefrontal cortex. This could also explain the preservation of long-range transhemispheric connections in the AD brain in an attempt to compensate for the lack of transmission from the anterior portion of the brain. Additionally, both brains had a highly connected module in the hindbrain, which included left and right inferior colliculus, left and right 2nd cerebellar lobule, left and right simple cerebellar lobule, left and right 3rd cerebellar lobule, left and right cerebellar peduncle, left and right 5th cerebellar lobule, and left and right cerebellar nuclear area. The functional role of cerebellar regions in the present amyloidosis mouse model is unclear and previous work in the TgCRND8 mouse model has not implicated this area. However, there is significant evidence in human imaging that sensorimotor cortical networks can map onto cerebellar cortical areas (Buckner et al., 2011). Hubs have been shown to be implicated in brain

disorders such as AD (Crossley et al., 2014) and thus a disconnected hub model may explain AD's effect on the brain rather than degradation of the hubs themselves.

REFERENCES

Akiyama, H., Harrop, R., McGeer, P. L., Peppard, R., & McGeer, E. G. (1989). Crossed cerebellar and uncrossed basal ganglia and thalarnic diaschisis in Alzheimer's disease. *Neurology*, *39*(4), 541–541. https://doi.org/10.1212/WNL.39.4.541

Bolding, K. A., Nagappan, S., Han, B.-X., Wang, F., & Franks, K. M. (2020). Recurrent circuitry is required to stabilize piriform cortex odor representations across brain states. *ELife*, *9*, e53125. https://doi.org/10.7554/eLife.53125

Buckner, R. L., Krienen, F. M., Castellanos, A., Diaz, J. C., & Yeo, B. T. T. (2011). The organization of the human cerebellum estimated by intrinsic functional connectivity. *Journal of Neurophysiology*, *106*(5), 2322–2345. https://doi.org/10.1152/jn.00339.2011

Busche, M. A., Eichhoff, G., Adelsberger, H., Abramowski, D., Wiederhold, K.-H., Haass, C., Staufenbiel, M., Konnerth, A., & Garaschuk, O. (2008). Clusters of Hyperactive Neurons Near Amyloid Plaques in a Mouse Model of Alzheimer's Disease. *Science*, *321*(5896), 1686–1689. https://doi.org/10.1126/science.1162844

Busche, M. A., & Konnerth, A. (2015). Neuronal hyperactivity – A key defect in Alzheimer's disease? *BioEssays*, *37*(6), 624–632. https://doi.org/10.1002/bies.201500004

Carrera, E., & Tononi, G. (2014). Diaschisis: Past, present, future. *Brain*, *137*(9), 2408–2422. https://doi.org/10.1093/brain/awu101

Crossley, N. A., Mechelli, A., Scott, J., Carletti, F., Fox, P. T., McGuire, P., & Bullmore, E. T. (2014). The hubs of the human connectome are generally implicated in the anatomy of brain disorders. *Brain*, *137*(8), 2382–2395. https://doi.org/10.1093/brain/awu132

Elman, J. A., Oh, H., Madison, C. M., Baker, S. L., Vogel, J. W., Marks, S. M., Crowley, S., O'Neil, J. P., & Jagust, W. J. (2014). Neural compensation in older people with brain amyloid-β deposition. *Nature Neuroscience*, *17*(10), 1316–1318. https://doi.org/10.1038/nn.3806

Fornito, A., Zalesky, A., & Breakspear, M. (2015). The connectomics of brain disorders. *Nature Reviews Neuroscience*, *16*(3), 159–172. https://doi.org/10.1038/nrn3901

Fournier, D. I., Monasch, R. R., Bucci, D. J., & Todd, T. P. (2020). Retrosplenial cortex damage impairs unimodal sensory preconditioning. *Behavioral Neuroscience*, *134*(3), 198–207. https://doi.org/10.1037/bne0000365

Grady, C. L., McIntosh, A. R., Beig, S., Keightley, M. L., Burian, H., & Black, S. E. (2003). Evidence from Functional Neuroimaging of a Compensatory Prefrontal Network in Alzheimer's Disease. *Journal of Neuroscience*, *23*(3), 986–993. https://doi.org/10.1523/JNEUROSCI.23-03-00986.2003

Klupp, E., Förster, S., Grimmer, T., Tahmasian, M., Yakushev, I., Sorg, C., Yousefi, B., & Drzezga, A. (2014). In Alzheimer's Disease, Hypometabolism in Low-Amyloid Brain Regions May Be a Functional Consequence of Pathologies in Connected Brain Regions. *Brain Connectivity*, *4*. https://doi.org/10.1089/brain.2013.0212

Kurucu, H., Colom-Cadena, M., Davies, C., Wilkins, L., King, D., Rose, J., Tzioras, M., Tulloch, J. H., Smith, C., & Spires-Jones, T. L. (2021). Inhibitory synapse loss and accumulation

of amyloid beta in inhibitory presynaptic terminals in Alzheimer's disease. *European Journal of Neurology*. https://doi.org/10.1111/ene.15043

Rubinov, M., & Sporns, O. (2010). Complex network measures of brain connectivity: Uses and interpretations. *NeuroImage*, *52*(3), 1059–1069. https://doi.org/10.1016/j.neuroimage.2009.10.003

Sperling, R. (2007). Functional MRI Studies of Associative Encoding in Normal Aging, Mild Cognitive Impairment, and Alzheimer's Disease. *Annals of the New York Academy of Sciences*, *1097*(1), 146–155. https://doi.org/10.1196/annals.1379.009

von Monakow, C. (1969). *Brain and Behaviour I: Mood, States and Mind* (K. Pribram, Ed.; pp. 27–36). Penguin Books.
